# Identification of transcription factor high accumulation DNA zones

**DOI:** 10.1186/s12859-023-05528-1

**Published:** 2023-10-20

**Authors:** Silvia Cascianelli, Gaia Ceddia, Alberto Marchesi, Marco Masseroli

**Affiliations:** 1 Dipartimento di Elettronica, Informazione e Bioingegneria, Politecnico di Milano, Via Ponzio 34/5, 20133 Milan, Italy; 2 Barcelona Supercomputing Center (BSC), 08034 Barcelona, Spain

**Keywords:** DNA high occupancy target zones, Transcription factor binding accumulation, Accumulation computation, Neighborhood-accounting moving window

## Abstract

**Background:**

Transcription factors (TF) play a crucial role in the regulation of gene transcription; alterations of their activity and binding to DNA areas are strongly involved in cancer and other disease onset and development. For proper biomedical investigation, it is hence essential to correctly trace TF dense DNA areas, having multiple bindings of distinct factors, and select DNA high occupancy target (HOT) zones, showing the highest accumulation of such bindings. Indeed, systematic and replicable analysis of HOT zones in a large variety of cells and tissues would allow further understanding of their characteristics and could clarify their functional role.

**Results:**

Here, we propose, thoroughly explain and discuss a full computational procedure to study in-depth DNA dense areas of transcription factor accumulation and identify HOT zones. This methodology, developed as a computationally efficient parametric algorithm implemented in an R/Bioconductor package, uses a systematic approach with two alternative methods to examine transcription factor bindings and provide comparative and fully-reproducible assessments. It offers different resolutions by introducing three distinct types of accumulation, which can analyze DNA from single-base to region-oriented levels, and a moving window, which can estimate the influence of the neighborhood for each DNA base under exam.

**Conclusions:**

We quantitatively assessed the full procedure by using our implemented software package, named TFHAZ, in two example applications of biological interest, proving its full reliability and relevance.

**Supplementary Information:**

The online version contains supplementary material available at 10.1186/s12859-023-05528-1.

## Background

Transcription factors (TFs) are proteins that bind the DNA at different sites, mainly at gene regulatory elements like promoters or enhancers. Often they interact with each other, or with co-factors, to form protein complexes that contribute to activating or repressing the bond between RNA polymerase and DNA [1, 2]. Thus, TFs play a crucial role in the regulation of gene transcription, and alterations of their activity are strongly involved in cancer onset and development, as well as in other disease settings [3, 4].

Next generation sequencing (NGS) techniques, mainly the chromatin immunoprecipitation followed by sequencing (ChIP-seq) [5, 6], made it possible to generate a large amount of reliable data regarding TF binding activity, which is increasingly available in public databases like GTRD [7], ReMap [8, 9], or those from well-known public research projects like the model organism encyclopedia of DNA elements (modENCODE) [10] and the encyclopedia of DNA elements (ENCODE) [11]. The genomic regions identified with ChIP-seq experiments are characterized by few direct TF-DNA interactions, or by protein-protein interactions of TFs with other regulators (other TFs or co-factors), or even by unspecific TF-DNA binding [2]. Many studies of the last decade focused on *Caenorhabditis elegans* [12–14], *Drosophila melanogaster* [10, 15–18] and *Homo Sapiens* [1, 3, 14, 19, 20], showed that the majority of the identified binding regions are low-occupancy sites, bound by one or few different TFs and enriched of specific motifs recognized only by the bound TFs, suggesting direct DNA binding. However, they also highlighted a still relevant number of DNA sites that are bound by many clustered TFs, usually without showing sequence-specific binding motifs: this indicated unlikely direct DNA binding and suggested that in these DNA areas TFs are enrolled non-specifically or through protein-protein interactions [2]. Because of the accumulation of non-specific bindings with different TFs, such DNA areas are commonly indicated as *dense* zones, and among them, the High Occupancy Target (*HOT*) zones are those exceeding a defined high number of bindings [1].

Targeted investigations of dense zones, occupied by many different TFs, are crucial for the comprehension of the mechanisms of gene expression regulation and of their alteration in pathological conditions, towards a deeper understanding of disease biology, diagnosis and therapeutic options [3, 4]. Some recent studies aimed at identifying specifically direct TF-DNA interaction events (e.g., [2]), while some others addressed the task of tracing and characterizing HOT zones (e.g., [21]). These latter ones indicated HOT zones as active genomic elements showing peculiarities associated with open chromatin, such as decreased nucleosome density and increased nucleosome turnover [3, 10, 12, 14, 17], and showed that HOT zones mostly match promotorial regions enriched for CpG islands in different organisms (e.g., [14]). Furthermore, they highlighted that genes proximal to such regions are usually housekeeping genes widely and stably expressed across multiple cell types [14, 21]; however, at locations enriched for disease-risk variants, HOT zones have also been associated with cell development, differentiation and oncogenesis [3, 4, 22].

Although many efforts have been made to identify the genome-wide occupancy profiles of a large number of TFs, the interpretation of the functional role of HOT zones is still unclear, both in physiological and diseased conditions, especially concerning how multiple TF interactions in such regions contribute combinatorially to the transcription regulation. This challenging investigation has been made more complex due to differences in the definition, identification and characterization of TF-dense and HOT zones [14, 21, 22]. Methods used for the identification of HOT zones mainly apply on aligned reads from NGS ChIP-seq experiments for multiple TFs; they extend previous peak-calling algorithms to the parallel analysis of multiple samples [1, 3], computing a combined signal of all the samples from which HOT zones are extracted. Conversely, other methods consider DNA enriched regions representing ChIP-seq signal peaks of every single TF under exam; then, they combine them in different ways to associate each DNA zone with a complexity score that represents the number of distinct TFs bound to it [10, 19, 23, 24]. Also, in these cases, DNA zones with a TF co-binding score greater than a certain threshold are defined as HOT, whereas further score intervals can be used to define other types of genomic regions, like WARM or COLD zones, with intermediate or opposite characteristics compared to HOT zones [10].

Overall, most of the methods used in the literature to identify HOT zones are complex and/or difficult to replicate due to incompleteness of their description, lack of implementation details, or absence of the input data from which HOT zones are obtained. Moreover, when an implementation is provided, its use is often not intuitive or easy to apply in a comparative analysis, especially on already processed public data [1]. However, it is essential to correctly trace TF-dense DNA areas and be able to systematically analyze HOT zones in DNA of a large variety of cells and tissues: this would allow a better understanding of their characteristics and shed light on their still ambiguous functional role.

Here, we present our methodology, developed as a computationally efficient parametric algorithm and implemented in an R/Bioconductor [25, 26] package. It uses a systematic procedure with two alternative methods to examine and evaluate TF bindings over DNA sequences as to offer meaningful comparative and fully-reproducible assessments. Even more importantly, it can innovatively provide evaluations with different levels of detail and resolution: indeed, it respectively introduces three distinct types of accumulation measures and the use of a moving window as to analyze DNA from single-base to wider areas, estimating the influence of the neighborhood for each base. Our goal was to develop and make available a novel, thoroughly explained procedure to study in-depth DNA zones dense with TF accumulation and identify HOT zones. Thus, we implemented this method in an open-source and fully-described R/Bioconductor package, called TFHAZ [27], i.e., Transcription Factor High Accumulation Zones, to provide researchers with a ready-to-use software. TFHAZ performs the analysis of TF accumulation in DNA regions entirely and efficiently without the need for high-performance computing infrastructures. We comparatively assessed the proposed procedure using both synthetic data to better explain its algorithm and computational methods, and real data from two example use cases of biological interest to prove its relevance and practical usefulness. The methodology is innovative, well-understandable and also completely reproducible. It enables any scientist to identify HOT zones based on the dataset of TF binding regions of interest, following the most suitable strategy of accumulation measure and search method based on the specific study goals or comparing different strategies and resolutions to strengthen the obtained results.

## Implementation

In the following subsections, we comprehensively present our TFHAZ R/Bioconductor package and the original methodology we developed and implemented in it. Notably, we highlight its different analytical alternatives, input data settings and main strengths: three quantification strategies to compute accumulation from DNA single-base to region-oriented exploration, two methods to identify the HOT zones, and the possibility of using a moving window strategy to account for the influence of the accumulation of DNA bases close to that/those under exam.

### TFHAZ R/bioconductor package

With the aim of providing ready-to-use software to apply our proposed search methodology for HOT DNA zones, we developed a computationally efficient R/Bioconductor package named TFHAZ (Transcription Factor High Accumulation Zones) [27]. It allows users to easily find DNA zones of high accumulation from datasets containing the genomic positions of TF binding regions. Input data must be loaded as a GRanges object, a widely used R/Bioconductor data structure with a section for genomic ranges where TF binding regions are listed and a metadata section where the name of the bound TF is annotated for each region. Note that *IRanges*, *GenomicRanges* and *GenomicFeatures* [28] packages offering this kind of scalable data structure are the core of the R/Bioconductor infrastructure for handling genomic data. They also provide efficient functions for data extraction and range-based operations, including coverage calculation. This latter one is used in TFHAZ to compute the accumulation vector, modelled as a run-length-encoded (RLE) object, which is a compact representation for very long vectors fully supported by *IRanges* functions. Hence, TFHAZ is fully integrated with existing R/Bioconductor functions and data structures, guaranteeing effective treatment and efficient processing of the considered genomic data, and interoperability with other R/Bioconductor packages (e.g., [28–30]).

TFHAZ R/Bioconductor package is freely available both in the official Bioconductor release (currently, version 3.16)^1^ and on a GitHub repository^2^ together with its complete documentation and its vignette. In its first five years, TFHAZ has been progressively enhanced, and overall it counts more than 5,500 downloads by more than 2,200 distinct IPs from Bioconductor only. TFHAZ is easy-to-use thanks to its clear documentation and vignette: these latter allow exploring all the package functionalities along with some examples of possible usage. Taking the TF binding regions of interest (as the ones extracted from ChIP-seq data) as input, TFHAZ offers functions for the computation of the different accumulation types, for the search of TF-dense DNA zones and the identification of the HOT ones. Additionally, it provides evaluation and plotting functions to compare the results obtained with different moving window sizes, accumulation types and threshold values. Specifically, to identify DNA HOT zones based on the computed accumulation vector, its *high_accumulation_zones()* function allows specifying the moving window size, the method to be used (*binding region* or *overlap*), and the desired thresholding procedure. Furthermore, the resulting HOT zones are returned together with the min, max, mean, median and standard deviation of their lengths.

### Methodology

**Fig. 1:**
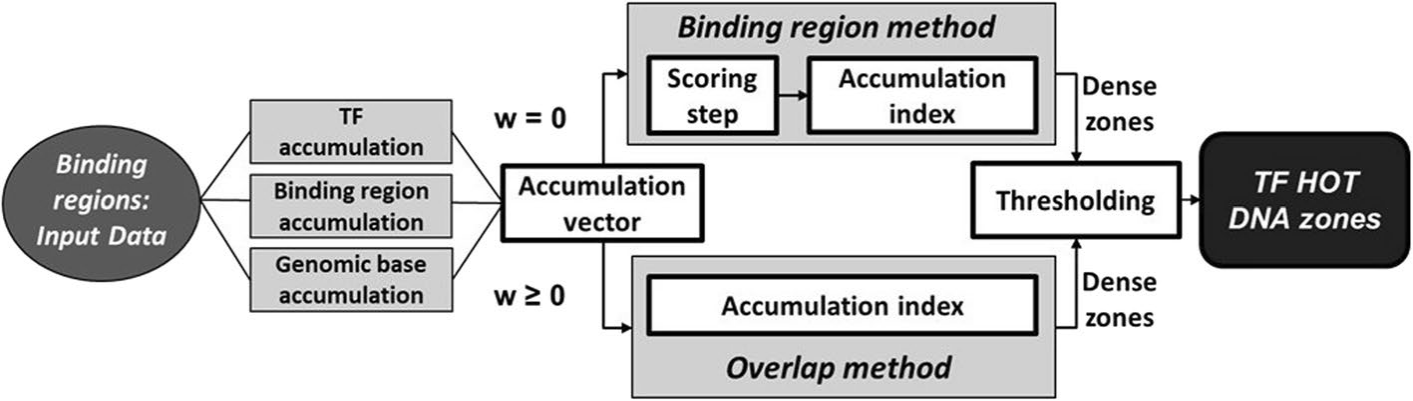
Workflow for the identification of high accumulation DNA zones, starting from input binding regions and using two alternative approaches: the *Binding region* or the *Overlap* method

In Fig. 1, we schematically illustrate our proposed procedure to find HOT zones implemented in the TFHAZ package. A dense zone (potentially HOT) is here defined as a DNA area in which a high number of different TFs bind, as summarized by its corresponding accumulation index; when this index exceeds a given threshold value, the dense zone is considered a HOT zone. This definition clarifies how to distinguish HOT zones from other TF-dense zones and leaves open the alternatives for computing the decisive accumulation index and threshold, as well as for the choice of the areas of interest between entire input binding regions or more specific subsets of DNA bases. Indeed, our procedure offers innovative ways to explore DNA areas of different sizes, also providing local single-base evaluation of TF-DNA interactions and three alternative quantification strategies to compute accumulation. Furthermore, we formalize two different methods to trace HOT zones, named *binding region* and *overlap* methods, as described in “Identification of DNA HOT zones” section. Such methods can work on a single chromosome as well as on the entire genome (based also on the ChIP-seq data provided as input), and are included in a systematic and fully-reproducible procedure for searching HOT zones.

The *binding region* method (top panel in Fig. 1), among all input binding regions, identifies those with a total number of different TFs greater than a computed threshold. Instead, the *overlap* method (bottom panel in Fig. 1), starting from the local evaluation of each DNA base under exam, finds contiguous bases having a number of overlapping bindings greater than a computed threshold. Regardless of the chosen method, the procedure starts with a local evaluation of the accumulation on every DNA base under analysis as to construct an accumulation vector. This can be obtained from ChIP-seq TF binding region data using the *TF accumulation*, the *binding region accumulation*, or the genomic *base accumulation* strategy alternatively, as detailed in “Accumulation types” section. Additionally, the accumulation value of each base can be influenced by the accumulation values of the bases on its neighborhood, according to the use of a moving window innovatively introduced for this purpose, as illustrated in “Moving window” section. This allows exploring different granularities of analysis together with distinct accumulation types. Local accumulation values are then aggregated to associate each zone of interest with an accumulation index. The *binding region* method focuses on the input binding regions (each bound by at least one TF) and requires an intermediate scoring step to compute region accumulation indexes. Conversely, the *overlap* method extracts as regions of interest all the sets of contiguous bases with the same local accumulation value: this simply becomes the accumulation index of the corresponding region. The procedure ends with a thresholding step, used to distinguish HOT zones from dense ones with a lower accumulation index; alternative options to compute a proper threshold are discussed in “Thresholding procedures” section.

**Fig. 2:**
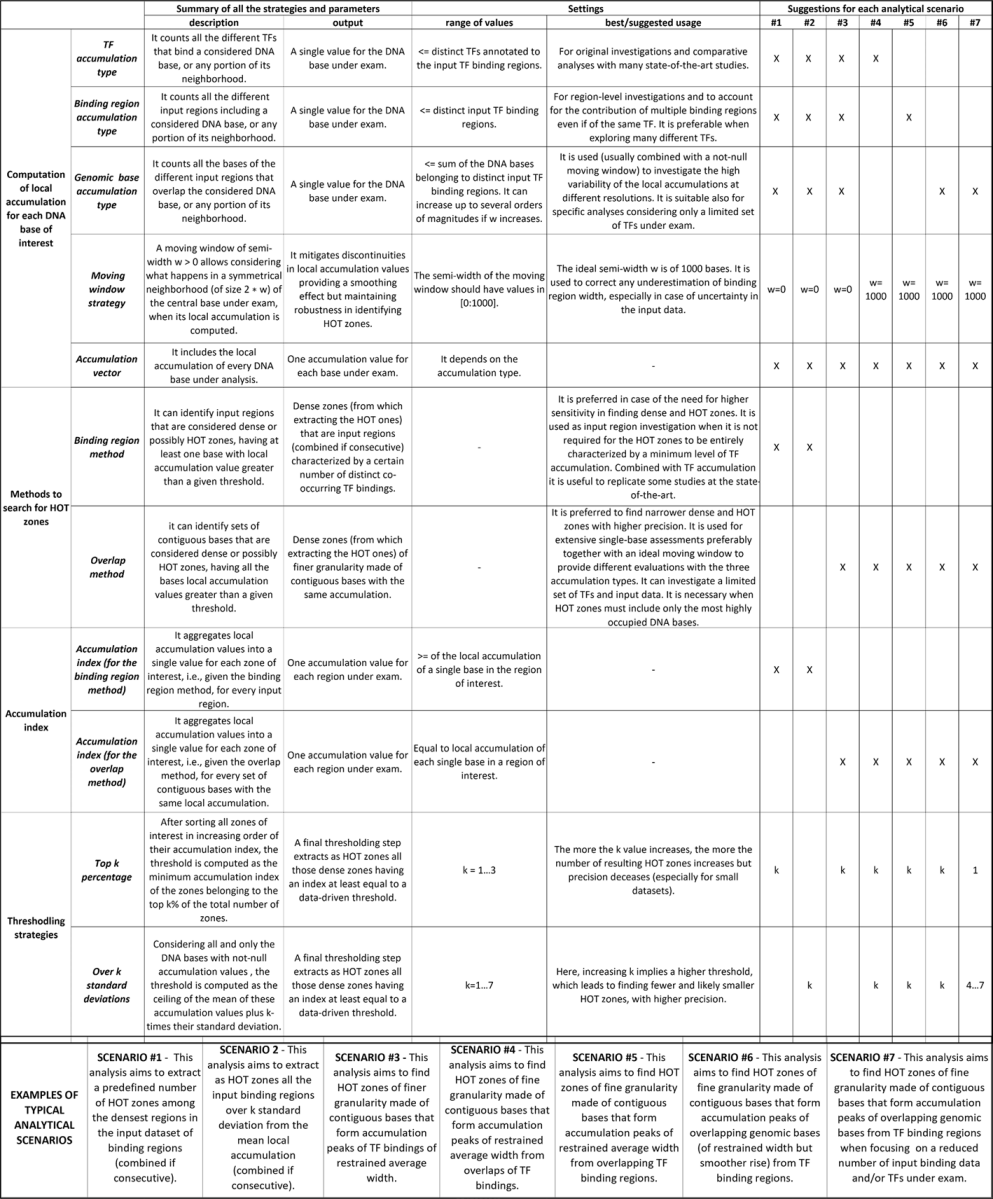
Overview of the strategies and parameters included in our methodology, and, at the bottom of the Figure, brief descriptions of 7 typical analytical scenarios for users. Together with the presentation and with some usage details for every strategy/parameter, advised settings are indicated on Figure right, considering each of the 7 analytical scenarios separately

In Fig. 2, we briefly recap all the strategies and parameters of our proposed methodology and indicate suggested choices for a series of typical analytical scenarios. Indeed, suitable parameter values and methodological options depend on the analysis intents and on the required output for following investigations. Factors such as the need for dense regions of bigger width rather than narrower peaks (e.g., using or not a moving window and adjusting the stringency of the thresholding), the trade-off between sensitivity and precision in recognizing HOT zones (e.g., using the *binding region* or the * overlap* method, respectively), the selection of a given portion of zones with higher density (e.g., using *top k percentage* thresholding) rather than of actual outlier zones (e.g., using an *over k standard deviations* thresholding) are decisive in guiding user choices. HOT zones can be retrieved following a specific setting or comparing alternative adequate options to strengthen the obtained results. Yet, at the state-of-the-art there are no absolute criteria to define the best HOT zones since there are no definitive sets of true HOT zones to track, nor a well-recognized gold standard to trace them. Thus, we used some comparative evaluation criteria to assess the reliability of the results found by our methodology, which emerged as crucial to offer a standardized approach ensuring complete repeatability but also flexibility to adapt to different analytical scenarios. Our HOT zones demonstrated both a solid core of concordant results between our two defined search methods (when both are adequate for a given analysis), and impressive coherence with existing studies, like [14], or with databases including comprehensive regulatory hotspot annotations, like [8, 9]; furthermore, they reflect known characteristics of the HOT zones, like enrichment in promoters, CpG islands, and promotorial CpG islands.

#### Input data settings

As input data, we consider all the TF binding regions organized within a matrix, where each row specifies a binding region with its genomic location and the name of the bound TF. This kind of data mostly comes from ChIP-seq narrow peaks. The first four columns of an input matrix represent the genomic coordinates of each binding region (i.e., the chromosome, the start and end positions, and the strand, respectively), while an additional fifth column hosts the corresponding TF. As for genomic coordinates, we consider a 1-base inclusive coordinate system: it directly numbers every DNA base (instead of specifying coordinates that flank each base) and includes in the analysis also the extreme bases at the start and end positions of the considered regions.

#### Moving window

ChIP-seq experiments generate millions of short reads that are usually retained only if they match unique locations (uni-reads) once mapped to the reference genome. This practice is mostly not harmful because many uni-reads are adjacent to discarded multi-reads and allow identifying an underlying peak of a binding site [31]. Yet, this can lead to a partial underestimate of the occupancy of binding regions, which may appear sparser and with contiguous bases much less homogeneous than they really are. To face this issue, a moving window mode was used to innovatively introduce the concept of neighborhood in the search for HOT zones. During the local analysis, performed one base at a time, the accumulation value of each base is computed considering what happens not only in the base itself but also in its symmetrical neighborhood of size $2*w$. Indeed, a moving window of semi-width *w* is centered on each DNA base under exam. The *w* value is an input parameter of this algorithm to search for dense zones; its variation offers multi-scale analyses with different resolutions in the local investigation of the binding regions.

A not-null moving window (i.e., of semi-width $w \ne 0$) allows capturing differences based on the chosen accumulation type, as discussed in the following “Accumulation types” section. Also, it mitigates the local discontinuities that clearly emerge in its absence, each time that consecutive bases have very different accumulation values. The *smoothing* effect grows as the moving window semi-width increases since the accumulation value of each base is more influenced by farther bases. Yet, too high values of *w* can lead to the aggregation of very distant bases and provide undesired fake artefacts on dense zones. Overall, the analysis detailed in Additional file 1: Section S1.1 indicates 1000 bases as the ideal semi-width of a moving window able to smooth local discontinuities without aggregating too far bases.

#### Accumulation types

The procedure computes local accumulation values, which are stored within an accumulation vector where each position refers to a specific DNA base. These values are obtained according to one of the following quantification strategies.

1. *TF accumulation* returns the number of distinct input TFs that bind the considered DNA base *b*, or any portion of its neighborhood (of amplitude $2 * w { + 1}$ centered on the base, if $w > 0$), i.e.,:
  $$\begin{aligned} TF_{acc} = \sum _{t=1}^{n_{TF}}t: \exists _{i=b-w}^{b+w} \left\{ t(i) \ne 0 \right\} \end{aligned}$$
  where $n_{TF}$ is the number of distinct TFs, *i* spans all over the DNA bases in the neighborood of *b* and $t(i) \ne 0$ denotes that a given TF *t* binds the base *i*.
2. binding *region accumulation* returns the number of all the different input binding regions, for either new or already considered TFs, that include the examined DNA base *b*, or any portion of its neighborhood (if $w > 0$), i.e.,:
  $$\begin{aligned} BR_{acc} = \sum _{r=1}^{n_{IR}}r: \left\{ r \cap (b\pm w) \ne 0 \right\} \end{aligned}$$
  where $n_{IR}$ is the number of distinct input binding regions, and $r \cap (b\pm w)$ is the intersection between a given input region *r* and the neighborood of the base *b* under exam.
3. genomic *base accumulation* returns the amount of DNA bases from all the different input TF binding regions that overlap the considered DNA base *b*, or its neighborhood (if $w > 0$), i.e.,:
  $$\begin{aligned} GB_{acc} = \sum _{r=1}^{n_{IR}}\sum _{d=1}^{n_{r}}d: \left\{ d \cap (b\pm w) \ne 0 \right\} \end{aligned}$$
  where $n_{IR}$ is the number of distinct input binding regions, $n_{r}$ is the number of bases of a given input region *r*, and $d \cap (b\pm w)$ is the intersection between a single DNA base *d* from the input region *r* and the neighborood of the base *b* under exam.

Without considering any neighborhood (i.e., for $w = 0$), the results of the three strategies are equal as long as no overlapping binding regions for the same transcription factor are present in the input data (as it usually is). Just in this latter case, a DNA base in the overlapping area of more binding regions for the same transcription factor has its *region* and *base accumulation* values equal and greater than its *TF accumulation* value.

**Fig. 3:**
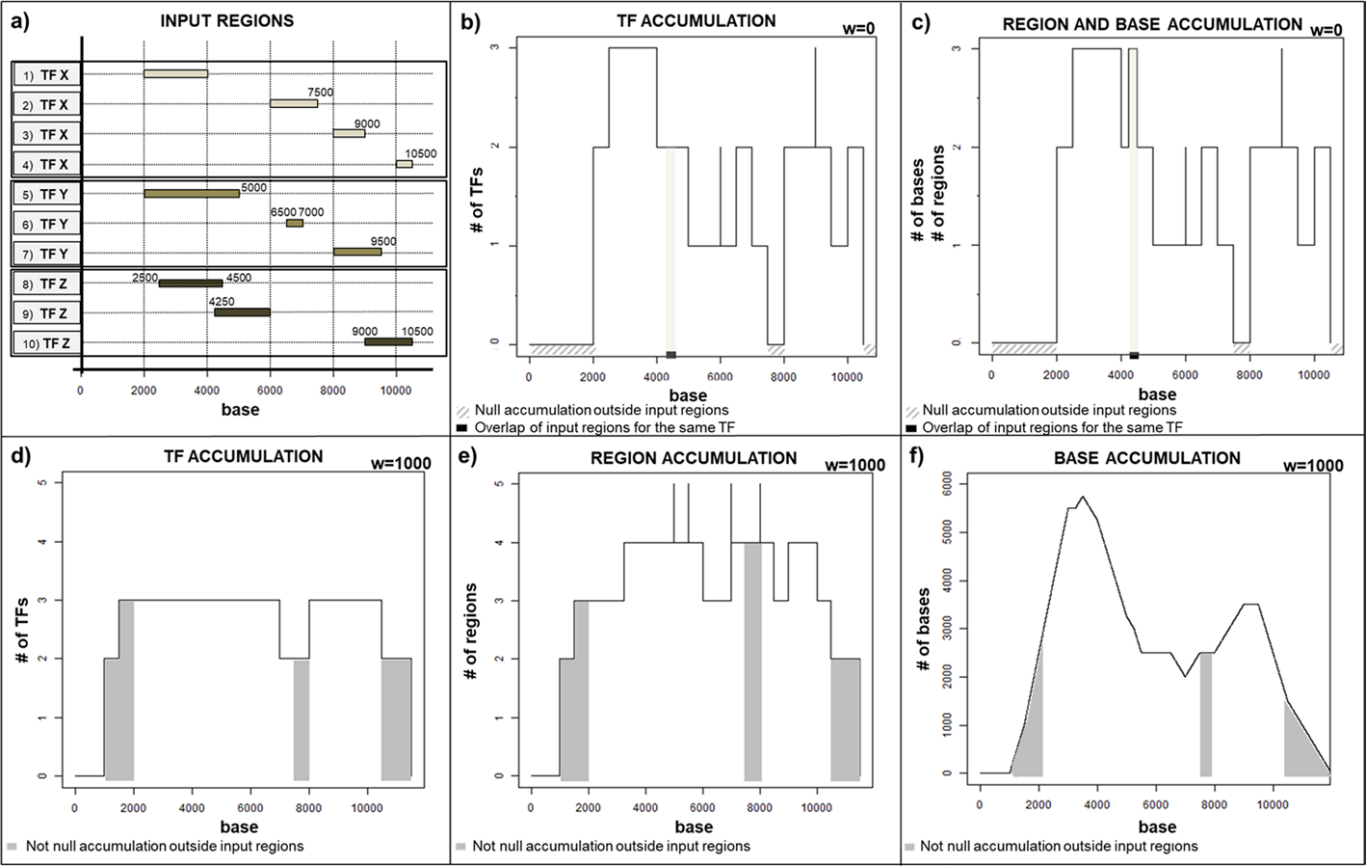
Accumulation values of an example dataset with 10 input transcription factor (TF) binding regions of three different TFs X, Y and Z (**a**). When considering a null moving window ($w = 0$) (**b**, **c**), the *TF accumulation* (**b**) is different from the *region accumulation* and *base accumulation* (**c**), due to the overlap of input binding regions for the same TF starting at position 4250. For an ideal moving window of 1000 bases of semi-width ($w = 1000$), each of the three accumulation types shows well-differentiated values (**d**, **e**, **f**)

For an explanatory example, let us consider an input dataset with 10 input binding regions of 3 distinct TFs (X, Y, Z) as illustrated in Fig. 3a, where the eighth and ninth regions are both bound by the TF Z with a partial overlap in the coordinate range [4250, 4500]. Accordingly, for $w = 0$ all three accumulation types return the same values everywhere except in the range [4250, 4500]. There, the *TF accumulation* is equal to 2 (Fig. 3b) since both TF Y and TF Z (although within two distinct regions) bind there; instead, the *region* and *base accumulation* are equal to 3 in that range (Fig. 3c) because exactly 3 regions and 3 bases from all input regions occur in [4250, 4500] when such range is analyzed one base at a time (without any neighborhood).

Differences in the three accumulation types are appreciable when using a *w* value different from zero to calculate the accumulation values. In this case, not only all DNA bases of each input region *r*, but also those bases included within every pair of intervals starting from the extremes of each region *r* and extending outward it for *w* positions have a not-null accumulation value. This is clearly visible when comparing panels b, c (*w* = 0) and d, e, f (*w* = 1000) in Fig. 3. Each accumulation value is influenced by the considered neighborhood: based on the used accumulation type, this implies that additional TFs, input regions or bases can contribute to computing each local accumulation. The *region accumulation* of a base under exam increases each time the neighborhood of the base intercepts another input binding region; conversely, the *TF accumulation* does it only if another binding region of a different TF is intercepted. This can be noticed by comparing these two accumulation types for *w* = 1000 at position 3250 in panels d and e of Fig. 3: in panel e, the *region accumulation* becomes 4 since the moving window intercepts the starting base of another input region, the ninth, located in [4250, 6000]; yet, the TF of the ninth region is Z, which is already accounted for the *TF accumulation* in panel d because it also binds the eighth region, which includes the base at position 3250. Accordingly, the *TF accumulation* at position 3250 is 3, which is also the maximum possible value of the *TF accumulation* for a dataset including three different TFs.

However, the moving window is incisive mainly in computing the *base accumulation*, since its dynamic range of values can change several orders of magnitude as the window size increases. To better explain how *base accumulation* is affected, we can focus on a single input region, as in the cases represented and described in Additional file 1: Section S1.2. Obviously, when generalizing to the typical dataset with multiple TFs and binding regions, all input region bases intercepting the neighborhood of each position under exam are summed and contribute to computing the *base accumulation* value. This leads to obtain wider dense zones, where *base accumulation* rises more smoothly but reaches much higher values than for *TF* or *region accumulation*; this is evident even in our simple example data when comparing panel f of Fig. 3 with all the other reported cases.

#### Identification of DNA HOT zones

Two different methods have been defined to find DNA HOT zones, the *binding region* and *overlap* methods, which investigate as DNA bases of interest only those with a not-null accumulation value.

The *binding region* method, commonly used in the literature, identifies regions characterized by a certain number of co-occurring bindings of different TFs. Specifically, its regions of interest are the original input binding regions without any neighborhood: yet, the ones with at least one base of overlap are combined in a unique region. A scoring step is performed to move from the base-level evaluation of *TF accumulation* to a region-oriented evaluation. This scoring assigns each region *r* with an accumulation index, which is the number of different TFs that bind at least one base of *r*. Thus, this accumulation index is always equal to or greater than the maximum *TF accumulation* that we can obtain for a single base within *r*. Dense zones can be easily traced by selecting regions based on their accumulation index: a final thresholding step extracts as HOT zones all those dense zones having an index at least equal to a data-driven threshold, computed as detailed in “Thresholding procedures” section.

The *overlap* method focuses on DNA zones of finer granularity compared to the input regions as to identify contiguous bases with accumulation peaks. Given whichever accumulation type and moving window size, the accumulation vector is examined to extract zones made of contiguous bases having the same accumulation value: this value is inherited as the accumulation index for the specific DNA zone. Dense zones can be selected based on their accumulation index, while HOT zones are just those with an accumulation index not lower than a threshold value (see “Thresholding procedures” section).

Since the *overlap* method searches for contiguous bases of high accumulation and the accumulation index of the bases forms peaks, the so-identified HOT zones may be more in number but always of smaller dimension than those found with the *binding region* method. In addition, within dense zones obtained with the *overlap* method, each base has an accumulation value equal to the accumulation index value of its zone; consequently, HOT zones include only bases with local accumulations not lower than the considered threshold. Conversely, with the *binding region* method, typically, not all bases of any dense or HOT zone reach the same number of co-present TFs. Some bases may have a local accumulation value smaller than the accumulation index value of their zone and even equal to 1 only. Such main differences are clearly visible in Additional file 1: Fig. S3, where, for both *binding region* and *overlap* methods, the same threshold is used to identify HOT zones in the example dataset presented in Fig. 3. In Additional file 1: Fig. S3, all bases of a HOT zone that have accumulation lower than the threshold are shown in yellow; they are extracted only by the *binding region* approach. The *overlap* method instead avoids this issue, overcoming the region-level aggregation to compute the accumulation index used in the *binding region* method.

#### Thresholding procedures

A proper choice of the threshold needed to identify the DNA HOT zones is crucial. It may be defined arbitrarily, but data-driven thresholds, calculated based on the distribution of the dense zones and of their accumulation values as resulting from the described methodological steps, are warmly suggested. Specifically, two alternative types of thresholds are here following discussed: the *top* *k* *percentage* and the *over* *k* *standard deviations*, both related to the *k* parameter.

The *top* *k* *percentage* threshold is determined through a parametric generalization of a state-of-the-art approach to deal with different accumulation methods, resolution levels, and amounts of binding regions and TFs under exam. After sorting all zones of interest in increasing order of their accumulation index, the threshold is computed as the minimum accumulation index of the zones belonging to the top *k*% of the total number of zones. The more the *k* value increases, the more the number of resulting HOT zones increases.

The *over* *k* *standard deviations* threshold is instead defined by an alternative procedure, which obtains a threshold value based on the main statistical indicators of the distribution of the single-base accumulation value. Considering all and only the DNA bases with not-null accumulation values (regardless of the chosen accumulation type), the threshold is computed as the ceiling of the mean of these accumulation values plus *k*-times their standard deviation. Here, increasing *k* implies a higher threshold, which leads to finding fewer and likely smaller HOT zones.

## Results from example applications

To fully assess quantitatively the described computational procedure and its implementation in the TFHAZ package, as well as to comparatively discuss the best usage of all its methodological options, we used it in two example use cases of biological interest. The first one aims at identifying DNA HOT zones in bulk transcription factor binding data from many transcription factors and cell lines. Specifically, it quantitatively compares the results easily provided by the TFHAZ package with those previously published using the same data, showing the efficiency and reliability of TFHAZ. The second example is focused on the identification and evaluation of cell line conserved and exclusive DNA HOT zones in three different cell lines when considering a set of only six transcription factors. Thus, it shows the flexibility and efficacy of the procedure in a different, more typical scenario of analysis.

### DNA HOT zones from bulk transcription factor data

To provide an analytical evaluation and discussion of the implemented methodology to find DNA HOT zones, we performed a comparative analysis between the results obtained with the two described methods (*binding region* and *overlap*) and those reported in [14], also investigating the presence of HOT zones in specific DNA areas along the human genome. These include promoters and CpG islands, where a high number of TFs is expected, and CIS-regulatory modules (CRMs), i.e., peaks of regulatory hotspots traced by the ReMap project [8, 9] and currently provided within a publicly available comprehensive atlas [9].

#### Data and analytical workflow

In [14], the identification of HOT zones is performed on 612 samples regarding a considerable number of TFs (159) and cell lines (90). The authors defined the occupancy of each input region as the number of unique factors found binding inside it and, then, selected as HOT zones those regions ranked in the top 1% based on their occupancy value. For a direct comparison, we reconstructed the dataset used in that work based on the ENCODE ChIP-seq files for the human assembly hg19 that the authors reported. Yet, it was not possible to retrieve precisely the same data evaluated in that work; the data we could find available refer to the same number of samples, TFs and cell lines but have small differences in their content, e.g., 18,851 (0.16%) input regions more (Additional file 1: Section S3).

To search for HOT zones in such an entire dataset, we used the *TF accumulation* spanned over the whole genome, being this accumulation type the most similar to the occupancy definition in [14]. Both the *binding region* and *overlap* methods were evaluated, and the *top* *k* *percentage* threshold was used to extract the top 1% of the dense zones as HOT, like in [14]. Eventually, we also assessed the presence of HOT zones in promoters and in CpG islands, as well as the proportions of promotorial and CpG-enriched areas intersecting the HOT zones identified with the illustrated approaches, as described in Additional file 1: Section S3.

#### Comparative results

**Table 1:** Comparison of results for the 159 transcription factor (TF) dataset analysed by *TF accumulation* (TF acc)

Input regions	Moving window	Dense zones	Dense bases	Dense zone avg length	Max TF acc	Method to search for HOT zones	Max TF index	Threshold 1%	HOT zones	HOT bases	HOT zone avg length
11,677,623	–	737,151	NA	NA	123	[14]	138	58	7419	NA	NA
11,696,474	–	738,914	359,787,326	485.2	124	Binding region	138	58	7442	16,352,213	2197.3
11,696,474	w = 0	738,914	359,787,326	485.2	124	Overlap	124	63	3563	569,572	160.9
11,696,474	w = 1000	328,888	1,332,054,159	4075.0	134	Overlap	134	77	3253	5,209,955	1601.6

*avg* average, *NA* not available

In Table 1, we summarized the results obtained with the described alternative methodologies of TFHAZ compared with those from [14]. Though Chen and colleagues did not specify the use of region aggregation, we can notice that they identified 737,151 dense zones bound by 1 to 138 factors among their 11,677,623 input regions. This result is very similar to that obtained with the *binding region* method (which joins overlapping input regions) using *TF accumulation*: 738,914 dense zones, with a difference of only 1763 zones more (i.e., 0.24%, in line with the difference in input regions), and with the same max TF region index (138). Also, the HOT zones found with the *binding region* method are just 0.31% more than those in [14] (7442 vs. 7419), and the same top 1% threshold determined a value of 58 concomitant factors in both analyses. Such slight differences are perfectly reasonable considering that the data available that we could use are not exactly the same as originally used in [14].

Using the *overlap method* with $w = 0$ instead, we found a slightly higher top 1% threshold value (63) and, consequently, fewer HOT zones (3563 vs. 7442—47.88%) with, as expected, a much smaller average length (7.28%). Indeed, in this case, each identified HOT zone is made of contiguous DNA bases having a *TF accumulation* of at least 63 factors. Thus, the HOT zones found by the *overlap method* with $w = 0$ are completely contained within the HOT zones found by the *binding region* method but include only 3.5% of their bases, having the highest *TF accumulation* values. The marked reduction in HOT zone bases and in the average length is mitigated when using the *overlap* method with its ideal moving window ($w = 1000$). In this case, the average lengths of both dense and HOT zones were about ten times bigger than for $w = 0$, with the HOT zone average length being still lower than, but closer to (72.89%), the one found with the *binding region* method. Furthermore, although the number of dense zones halved (44.51%), the number of HOT zones remained stable (3253 vs. 3563—91.30%).

This clearly shows that the use of the moving window provides smoother evaluations, thanks to the contribution of the neighbour bases, maintaining robustness in the identification of the HOT zones (most HOT zones overlap with the ones found with $w = 0$), which are more precisely determined than with the *binding region* method. Coherent results can also be appreciated when comparing the *binding region* and *overlap* methods focusing on a single chromosome at a time, as we can see as an example in Additional file 1: Fig. S5 for chromosome 21.

#### Experimental evidences

The presented methods are systematic approaches that can be easily used through the TFHAZ package in a comparative way to trace HOT zones using different resolutions. When the *overlap* method is used without a moving window ($w = 0$), only very small regions corresponding to the accumulation peaks reach a TF accumulation value above the HOT zone threshold. Conversely, a moving window of 1000-base semi-width can correct possible underestimations of the binding region occupancy due to the experimental resolution, and mitigate the local discontinuities that cause the main differences between the *overlap* and *binding region* methods.

Thus, from our comparison, the *binding region* method should be preferred when focusing on region-level evaluations, particularly when not requiring HOT zones to be entirely characterized by a minimum level of TF accumulation. This formalizes an approach already in use [14], guaranteeing full reproducibility of the achieved results. The *overlap* method allows instead a more extensive analysis of the input data, focused on single-base assessments: these provide greater differentiation of the dense zones and allow retrieving as HOT zones only the most highly occupied contiguous DNA bases. Accordingly, when this is a requirement, the *overlap* method must be chosen, particularly if used together with an ideal moving window to attenuate the accumulation discontinuities introduced with the single-base assessments.

Regardless of their differences, both the *binding region* and *overlap* methods provide a remarkable core of concordant results: in the considered dataset, 3151 (96.86%) of the HOT zones found with the *overlap* method using $w = 1000$ intersect HOT zones obtained with the *binding region* method. While the *overlap* method improves the precision of the collected results, the *binding region* method is more focused on ensuring sensitivity. When compared with the ReMap CRM peaks of regulatory hotspots as a further evaluation criterion, 100% of the HOT zones resulting from both our *binding region* method and *overlap* method (either with or without ideal moving window) are overlapping with such relevant CRMs. Particularly, for the *overlap* method without moving window, which returns as HOT zones peaks of limited width but made of DNA bases with very high and continuous accumulation, 99.7% of the so-found HOT bases are completely included within ReMap peaks from the CRM atlas.

Our reference study [14] also highlighted that HOT zones in human genomes often lay in CpG-enriched promoters, suggesting that the high number of TFs bound to HOT zones may be a consequence of chromatin accessibility in CpG islands. Therefore, we further investigated the HOT zones found in DNA promotorial areas and CpG islands, as well as the promoters and CpG islands intersecting the HOT zones. Table 2 shows that many (more than 80% on average) of the found HOT zones are in CpG and promotorial areas. Both the described methods capture HOT zones showing a statistically significant association (Fisher test, *p* value < 2.2E−16) with promotorial and CpG-rich areas (Additional file 1: Tables S1 and S2).

**Table 2:** HOT zones identified by the *binding region* and *overlap* methods compared with known promotorial and CpG-enriched areas in the 159 TF dataset

Method to search for HOT zones	HOT zones	CpG islands	Promotorial regions	Promotorial CpG islands	HOT zones in CpG islands	HOT zones in promoters	HOT zones in promotorial CpG islands	CpG islands in HOT zones	Promoters in HOT zones
Binding region	7442	28,691	49,052	23,153	5958 (80.1%)	6064 (81.5%)	5549 (74.6%)	6335 (22.1%)	10,652 (21.7%)
Overlap (w = 0)	3563	28,691	49,052	23,153	2673 (75.0%)	2935 (84.4%)	2497 (70.1%)	2088 (7.3%)	3796 (7.7%)
Overlap (w = 1000)	3253	28,691	49,052	23,153	2518 (77.4%)	2818 (86.6%)	2361 (72.6%)	2523 (8.8%)	4816 (9.8%)

### Analysis of conserved and exclusive DNA HOT zones

To show the relevance and versatile usability of the described methods implemented in the TFHAZ software, we used it to identify TF high accumulation zones conserved in multiple cell lines and those exclusive of just a cell line.

#### Data and analytical workflow

We focused on three cell lines from the ENCODE project [11], i.e., H1-hESC—an embryonic stem cell line, K562—a myelogenous leukemia cell line, and MCF-7—a human breast cancer cell line. From the GMQL repository [32], through its Web interface,^3^ we extracted all TF binding region data regarding the only six TFs (CTCF, JUN, MAFK, MYC, NRF1, RFX5) whose ChIP-seq data are available in the latest version of the ENCODE project for all three cell lines. Details about the performed data processing are available in Additional file 1: Section S4.

With the TFHAZ software, we searched HOT zones of each cell line using the *overlap* and the *binding region* methods with the three types of accumulation for both a null and an ideal moving window, and we applied the two thresholding strategies (*top* *k* *percentage* and *over* *k* *standard deviations*), with different *k* values, alternatively. From this comparison, we noticed that when a small number of investigated TFs limits the accumulation values, the *binding region* method struggles with identifying actual HOT zones, regardless of the used thresholding option. Conversely, the *overlap* method also works well in this analytical scenario (see Additional file 1: Table S3). Notably, its use with *base accumulation* values obtained with an ideal moving window is preferable when examining a reduced set of TFs. In this case, the accumulation dynamic range is widely enlarged thanks to the considered neighborhood and the contribution of single DNA bases: this allows better distinguishing the most highly TF-targeted DNA zones from other dense zones. Furthermore, with the *overlap* method, the computed data-driven threshold is more sensitive to the chosen thresholding strategy and *k* value (see Additional file 1: Table S3); thus, this latter can be better calibrated to achieve the desired analytical resolution with the *over* *k* *standard deviations* strategy, especially when working on datasets of more limited sizes, as in this example. Therefore, ultimately we compared the DNA zones of each cell line that are highly occupied by the six TFs, as obtained with the just mentioned preferred combination of searching options.

**Table 3:** Comparison of results for 3 cell lines analysed by *base accumulation* with an ideal moving window

Cell line	Input regions	Moving window	Method to search for HOT zones	Threshold		HOT zones	HOT bases	HOT zone avg length	HOT zone avg length standard dev	Conserved HOT zones	Exclusive HOT zones
				Type	Value						
H1-hESC	81,006	w = 1000	Overlap	4 STD	806	1092	1,434,460	1313.61	545.76	124 (11.4%)	687 (62.9%)
K562	163,444	w = 1000	Overlap	4 STD	2478	1739	1,529,492	879.52	500.84	122 (7.0%)	1,211 (69.6%)
MCF-7	164,108	w = 1000	Overlap	5 STD	1858	1258	1,264,581	1005.23	658.57	121 (9.6%)	835 (66.4%)

For each cell line, we used the *overlap* method with an *over* *k* *standard deviations* (k STD) threshold to find HOT zones, and then extracted those zones conserved among all the three cell lines, or exclusive of each cell line

*avg* average, *dev* deviation

#### Comparative results and experimental evidence

In Table 3, we reported the results of the *overlap* method when using the *base accumulation* with an ideal moving window and a tuned *over* *k* *standard deviations* threshold. After discriminating cell line-exclusive HOT zones and conserved ones among all our three cell lines (i.e., overlapping, although partially, with at least a HOT zone of both the other two cell lines), we traced the genes associated only with HOT zones in just one cell line of interest (as specified in Additional file 1: Section S4). Each gene list was then independently evaluated using functional enrichment analysis (Additional file 1: Section S4) to assess any significance in gene ontology (GO) and/or pathway annotations. Some enriched terms (e.g., the GO molecular function term *protein binding* and biological process term *regulation of cellular process*) are shared across the three gene lists, as expected; some other terms are instead specific for each cell line and may be worthy of further investigation.

In particular, the analysis of the H1-hESC gene list showed the enrichment of the *Basal transcription factors* and *Wnt signalling pathway* (from KEGG [33]), which regulate crucial aspects of cell differentiation and organogenesis during embryonic development [34]. From the analysis of the K562 gene list, we noticed the enrichment of the *Glutathione metabolism* (from KEGG), which plays a key role in antioxidant defence and regulation of gene expression, cell proliferation, cytokine production and immune response [35]. Eventually, the analysis of the MCF-7 gene list reported the enrichment of the pathways devoted to the *Transport of nucleotide sugars* (from Reactome [36]) and to the *Choline metabolism in cancer* (from KEGG): deficiency in nucleotide sugar transporters is known to be involved in tumour metastasis [37], while deregulation of choline metabolism is one of the most consistent hallmarks of cancer [38].

Overall, our methodology allows researchers to find interesting and reliable outcomes not only when inspecting the complete spectrum of binding regions and corresponding TFs, but also when addressing targeted investigations to compare different biological scenarios focusing on a limited set of TFs.

## Conclusions

The here proposed procedure to investigate TF-dense zones makes use of three distinct types of accumulation and an innovative moving window approach to assess the influence of the neighborhood of each DNA base. Most importantly, to identify HOT zones, it provides two alternative methods that support different DNA exploration resolutions, from single base to region-oriented.

Beyond fully describing the methodology implemented in our TFHAZ R/Bioconductor package, we provided two noteworthy examples of its biological utility and reliability. The obtained results were compared with each other and with those in the literature: TF dense and HOT zones of the DNA were analysed in terms of occupied DNA bases, average lengths and distributions along the genome. Localization of HOT zones in specific DNA areas was evaluated, and their high concentration in promoters and CpG islands was confirmed. Additionally, when considering different cell lines and the same set of TFs of interest, the results of the approach showed that cell line-specific HOT zones confirm the expected enrichment of protein binding and regulatory activities, besides the significant association with some particular traits concordant with the cell line characteristics.

Precise and reproducible identification of DNA TF-dense areas and selection of the HOT zones with the highest accumulation of TF bindings is paramount for a complete comprehension of these DNA regulatory regions. TFHAZ functionalities are versatile and valuable to perform such investigations in the R/Bioconductor environment and to obtain fully reproducible results and comparative assessments considering different resolutions, from DNA single-base to region-oriented explorations.

## Availability and requirements

- Project name: TFHAZ
- Project home page: on GitHub: https://github.com/DEIB-GECO/TFHAZ on Bioconductor: https://www.bioconductor.org/packages/release/bioc/html/TFHAZ.html
- Operating system(s): Platform independent
- Programming languages: R
- Other requirements: R ($$\ge$$ 3.5.0)
- License: Artistic$$-$$2.0
- Any restrictions to use by non-academics: None

### Supplementary Information

**Additional file 1.**  Supplementary Material.

## Data Availability

TFHAZ package, documentation and code are freely available at https://www.bioconductor.org/packages/release/bioc/html/TFHAZ.html and https://github.com/DEIB-GECO/TFHAZ. This resource will also be soon available to users as a service of the ELIXIR-IT portfolio. On GitHub, we also provide a BED file containing the HOT regions resulting from global analysis (as here in the first example use case) of recent ENCODE ChIP-seq Narrow peak data extracted from our GMQL public repository [32]. All the data underlying this article were derived from sources in the public domain:  http://www.gmql.eu/ (GMQL [32] through the available Guest Login) and  https://personal.broadinstitute.org/anshul/projects/encode/rawdata/peaks_spp/mar2012/distinct/idrOptimalBlackListFilt/.

## Abbreviations

ChIP-seq: Chromatin immuno-precipitation followed by sequencing
CpG: Palindromic DNA stretches having repeated cytosine and guanine nucleotides with the “p” representing the linking phosphate
DNA: Deoxyribonucleic acid
ENCODE: Encyclopedia of DNA elements
GMQL: GenoMetric query language
GO: Gene ontology
hg19: Human genome reference version 19
HOT: High occupancy target
KEGG: Kyoto encyclopedia of genes and genomes
modENCODE: Model organism encyclopedia of DNA elements
NGS: Next generation sequencing
RLE: Run-length-encoded
STD: Standard deviation
TF: Transcription factor
TFHAZ: Transcription factor high accumulation zones

## Footnotes

1 https://bioconductor.org/packages/release/bioc/html/TFHAZ.html.

2 https://github.com/DEIB-GECO/TFHAZ.

3 http://www.gmql.eu/.

## References

[1] Foley JW, Sidow A (2013). Transcription-factor occupancy at HOT regions quantitatively predicts RNA polymerase recruitment in five human cell lines. BMC Genomics.

[2] Gheorghe M, Sandve GK, Khan A, Chèneby J, Ballester B, Mathelier A (2019). A map of direct TF-DNA interactions in the human genome. Nucleic Acids Res.

[3] Li H, Chen H, Liu F, Ren C, Wang S, Bo X (2015). Functional annotation of HOT regions in the human genome: implications for human disease and cancer. Sci Rep.

[4] Bushweller JH (2019). Targeting transcription factors in cancer—from undruggable to reality. Nat Rev Cancer.

[5] Johnson DS, Mortazavi A, Myers RM, Wold B (2007). Genome-wide mapping of in vivo protein-DNA interactions. Science.

[6] Landt SG, Marinov GK, Kundaje A, Kheradpour P, Pauli F, Batzoglou S (2012). ChIP-seq guidelines and practices of the ENCODE and modENCODE consortia. Genome Res.

[7] Yevshin I, Sharipov R, Valeev T, Kel A, Kolpakov F (2016). GTRD: a database of transcription factor binding sites identified by ChIP-seq experiments. Nucleic Acids Res.

[8] Chèneby J, Gheorghe M, Artufel M, Mathelier A, Ballester B (2018). ReMap 2018: an updated atlas of regulatory regions from an integrative analysis of DNA-binding ChIP-seq experiments. Nucleic Acids Res.

[9] Hammal F, de Langen P, Bergon A, Lopez F, Ballester B (2022). ReMap 2022: a database of Human, Mouse, Drosophila and Arabidopsis regulatory regions from an integrative analysis of DNA-binding sequencing experiments. Nucleic Acids Res.

[10] Roy S, Ernst J, Kharchenko PV, Kheradpour P, Negre N, Eaton ML (2010). Identification of functional elements and regulatory circuits by Drosophila modENCODE. Science.

[11] ENCODE Project Consortium, et al. An integrated encyclopedia of DNA elements in the human genome. Nature. 2012;489(7414):57. 10.1038/nature11247 PMC3439153 22955616

[12] Gerstein MB, Lu ZJ, Van Nostrand EL, Cheng C, Arshinoff BI, Liu T (2010). Integrative analysis of the Caenorhabditis elegans genome by the modENCODE project. Science.

[13] Van Nostrand EL, Kim SK (2013). Integrative analysis of *C. elegans* modENCODE ChIP-seq data sets to infer gene regulatory interactions. Genome Res.

[14] Chen RAJ, Stempor P, Down TA, Zeiser E, Feuer SK, Ahringer J (2014). Extreme HOT regions are CpG-dense promoters in *C. elegans* and humans. Genome Res.

[15] Moorman C, Sun LV, Wang J, de Wit E, Talhout W, Ward LD (2006). Hotspots of transcription factor colocalization in the genome of Drosophila melanogaster. Proc Natl Acad Sci.

[16] MacArthur S, Li XY, Li J, Brown JB, Chu HC, Zeng L (2009). Developmental roles of 21 Drosophila transcription factors are determined by quantitative differences in binding to an overlapping set of thousands of genomic regions. Genome Biol.

[17] Nègre N, Brown CD, Ma L, Bristow CA, Miller SW, Wagner U (2011). A cis-regulatory map of the Drosophila genome. Nature.

[18] Kvon EZ, Stampfel G, Yáñez-Cuna JO, Dickson BJ, Stark A (2012). HOT regions function as patterned developmental enhancers and have a distinct cis-regulatory signature. Genes Dev.

[19] Yip KY, Cheng C, Bhardwaj N, Brown JB, Leng J, Kundaje A (2012). Classification of human genomic regions based on experimentally determined binding sites of more than 100 transcription-related factors. Genome Biol.

[20] Yan J, Enge M, Whitington T, Dave K, Liu J, Sur I (2013). Transcription factor binding in human cells occurs in dense clusters formed around cohesin anchor sites. Cell.

[21] Wreczycka K, Franke V, Uyar B, Wurmus R, Bulut S, Tursun B (2019). HOT or not: examining the basis of high-occupancy target regions. Nucleic Acids Res.

[22] Li H, Liu F, Ren C, Bo X, Shu W (2016). Genome-wide identification and characterisation of HOT regions in the human genome. BMC Genomics.

[23] Chen H, Li H, Liu F, Zheng X, Wang S, Bo X (2015). An integrative analysis of TFBS-clustered regions reveals new transcriptional regulation models on the accessible chromatin landscape. Sci Rep.

[24] Mann FG, Van Nostrand EL, Friedland AE, Liu X, Kim SK (2016). Deactivation of the GATA transcription factor ELT-2 is a major driver of normal aging in *C. elegans*. PLoS Genet.

[25] R Core Team. R: a language and environment for statistical computing; 2020. Vienna, Austria. https://www.R-project.org/

[26] Gentleman RC, Carey VJ, Bates DM, Bolstad B, Dettling M, Dudoit S (2004). Bioconductor: open software development for computational biology and bioinformatics. Genome Biol.

[27] Marchesi A, Masseroli M. TFHAZ: transcription factor high accumulation zones; 2022. R/Bioconductor package version 1.18.0

[28] Lawrence M, Huber W, Pages H, Aboyoun P, Carlson M, Gentleman R (2013). Software for computing and annotating genomic ranges. PLoS Comput Biol.

[29] Huber W, Carey V, Gentleman R, et al. Orchestrating high-throughput genomic analysis with Bioconductor. Nat Methods. 2015;12:115–121. 10.1038/nmeth.3252 PMC4509590 25633503

[30] Pallotta S, Cascianelli S, Masseroli M. RGMQL: scalable and interoperable computing of heterogeneous omics big data and metadata in R/Bioconductor. BMC Bioinformatics. 2022;23(123). 10.1186/s12859-022-04648-4 PMC8991469 35392801

[31] Chung D, Kuan PF, Li B, Sanalkumar R, Liang K, Bresnick EH (2011). Discovering transcription factor binding sites in highly repetitive regions of genomes with multi-read analysis of ChIP-Seq data. PLoS Comput Biol.

[32] Masseroli M, Canakoglu A, Pinoli P, Kaitoua A, Gulino A, Horlova O (2019). Processing of big heterogeneous genomic datasets for tertiary analysis of Next Generation Sequencing data. Bioinformatics.

[33] Kanehisa M, Goto S (2000). KEGG: Kyoto encyclopedia of genes and genomes. Nucleic Acids Res.

[34] Sanz-Ezquerro JJ, Münsterberg AE, Stricker S (2017). Signaling pathways in embryonic development. Front Cell Dev Biol.

[35] Kennedy L, Sandhu JK, Harper ME, Cuperlovic-Culf M (2020). Role of glutathione in cancer: from mechanisms to therapies. Biomolecules.

[36] Gillespie M, Jassal B, Stephan R, Milacic M, Rothfels K, Senff-Ribeiro A (2022). The reactome pathway knowledgebase 2022. Nucleic Acids Res.

[37] Ishida N, Kawakita M (2004). Molecular physiology and pathology of the nucleotide sugar transporter family (SLC35). Pflugers Arch.

[38] Glunde K, Penet MF, Jiang L, Jacobs MA, Bhujwalla ZM (2015). Choline metabolism-based molecular diagnosis of cancer: an update. Expert Rev Mol Diagn.

