# Supplementary material for: Identification of transcription factor high accumulation DNA zones
Source: BMC Bioinformatics. 2023 Oct 20;24:395. doi: 10.1186/s12859-023-05528-1 (PMC10590011; doi:10.1186/s12859-023-05528-1)
Supplement: Supplementary file 1 — Additional file 1. Supplementary Material. [file 12859_2023_5528_MOESM1_ESM.pdf]

Supplementary material of "Identification of transcription factor high accumulation DNA zones"

Silvia Cascianelli[1], Gaia Ceddia[2], Alberto Marchesi[1], and Marco Masseroli[1]

[1]Dipartimento di Elettronica, Informazione e Bioingegneria, Politecnico di Milano, Milan, 20133 Italy
[2]Barcelona Supercomputing Center (BSC), Barcelona, 08034 Spain

S1 MOVING WINDOW

S1.1 Ideal size

We performed a genome-wide analysis to establish an ideal value of the moving window semi-width w. We took as a reference dataset the ENCODE ChIP-seq data of the Ishikawa human cell line of endometrial adenocarcinoma, which is widely used in basic research of biology and molecular science. This dataset contains 283,009 binding regions of 16 different transcription factors (TFs) and has a wide but manageable dimensionality for an extensive investigation considering all chromosomes. To find the ideal size of the moving window semi-width w, we did not optimize a scoring criterion, but we examined three aspects, considering increasing values of w from 0 to 100,000. The first two evaluations were the number and the mean length of the dense DNA zones found when varying the accumulation threshold, considering one w value at a time. Considering all the accumulation types and chromosomes (like in the example about chromosome 21 and TF accumulation, in Supplementary Figure 1.a and 1.b), the number of zones and their average length appeared quite consistent for all w up to 1000, even if this latter value can increase the dynamic range and enhance the local resolution of our analyses. This can be appreciated in Supplementary Figure 1, which reports as an example the number (a) and the mean length (b) of dense DNA zones on chromosome 21 for different TF accumulation threshold values, considering increasing values of semi-amplitude w from 0 to 100,000. For w = 0, 100 and 1000, the trend of the number of zones and their average length is very similar, while for higher values, it varies considerably. Another useful indication comes from our third evaluated aspect, i.e., the joint assessment of the number of dense zones and total DNA bases belonging to these zones when varying the window semi-width w. Supplementary Figure 1 (c) shows that, as expected, for increasing values of w, the number of dense zones decreases, while the number of total DNA bases belonging to these zones increases. Precisely, for $w = 1000$, both these measures show the greatest change in slope. For the following order of magnitude of w, there is instead a dramatic decrease in the number of dense zones (and an increase in the number of involved bases). Thus, w = 1000 mitigates local discontinuities without affecting the robustness of the analysis, whereas bigger values of w would instead merge too far bases. Therefore, from this analysis, 1000 bases appeared as the ideal choice for the window semi-width w, regardless of the chosen accumulation type.

Thus, a moving window of semi-width 1000 bases emerged as the ideal solution, being sufficiently wide to smooth the discontinuities in our local evaluations, but without aggregating too far bases.

S1.2 Influence on accumulation types

In the main article, we describe three types of accumulation that can be associated with each DNA base under exam: the *TF accumulation*, which counts the number of distinct TFs binding the base or its neighborhood (if $w > 0$); the *region accumulation*, which counts how many distinct input regions include the base or any portion of its neighborhood (if $w > 0$); and the *base accumulation*, which counts how many bases from all input regions overlap the considered base or its neighborhood (if $w > 0$). Relevant differences in the three accumulation types are appreciable when $w > 0$, as described in the main article.

Focusing on a single input region, we can better understand why mainly the *base accumulation* is affected by the neighborhood of the examined DNA base (i.e., by the use of a moving window) and its size

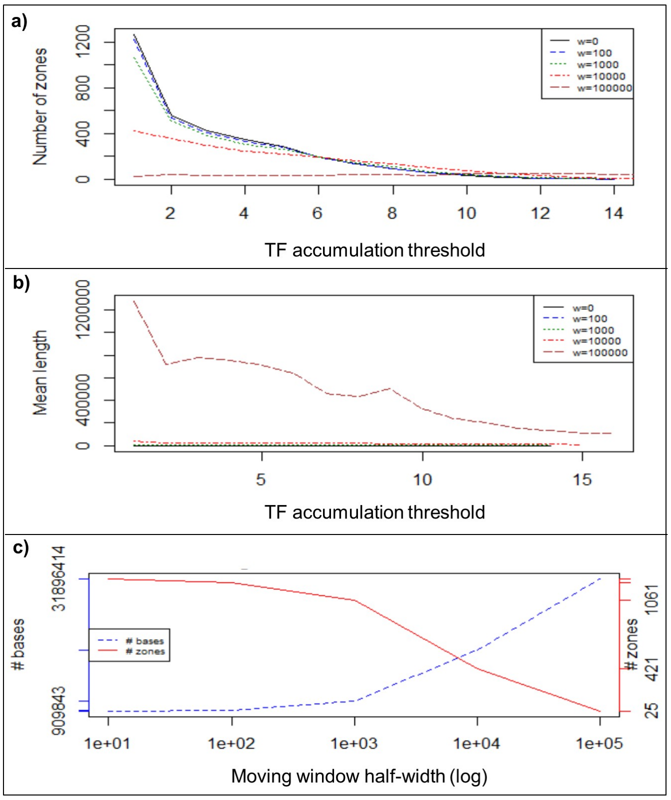

Supplementary Figure 1. Number of dense zones (a) and their mean length in base pairs (b) on human chromosome 21 based on different moving window semi-widths w, when considering increasing threshold values for the transcription factor (TF) accumulation. In (c), we can observe the decrease in the number of zones, and the increase of the involved bases belonging to these zones, when considering increasing moving window semi-widths with a minimum TF accumulation of 1.

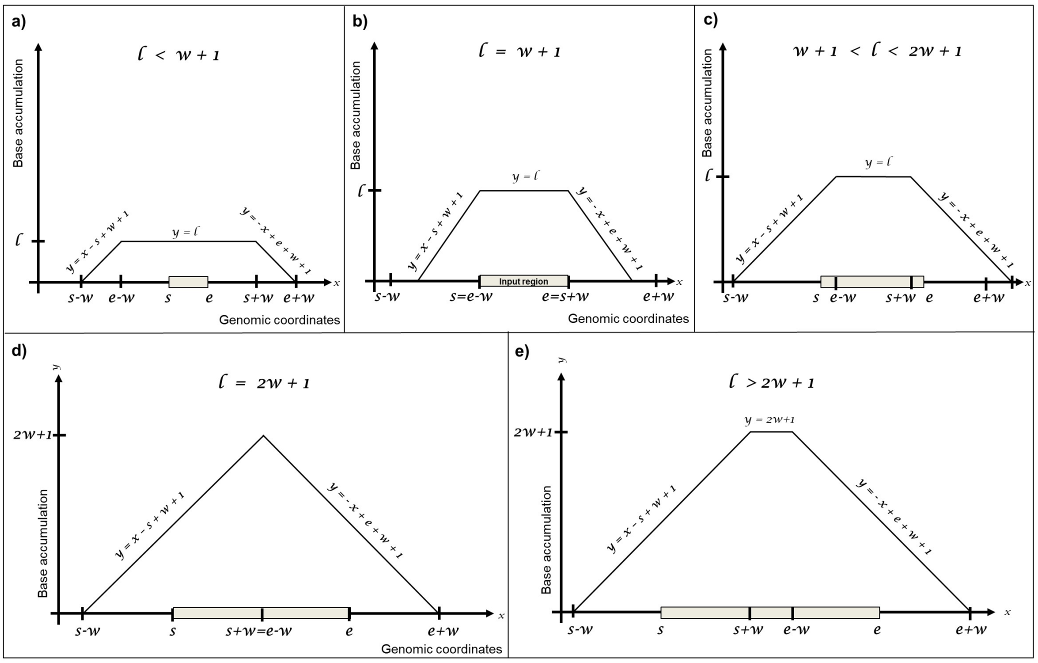

Supplementary Figure 2. *Base accumulation* of a single input binding region when using a moving window of semi-width w, considering different input region lengths. In the 1-base inclusive coordinate system, calling s the first base, e the last base and l the length of the input region: $l = e - s + 1$.

$(2 * w)$. Given the start s and end e positions of the region, its length l is equal to $e - s + 1$. In such a single

input region example, *TF accumulation* and *region accumulation* are both trivially equal to 1 for each DNA base going from $s-w$ until $e+w$, and equal to 0 otherwise. Conversely, as shown in Supplementary Figure 2, the maximum *base accumulation* value depends both on the region length and on the semi-width of the chosen neighborhood (w). A region with a length smaller than $2*w+1$ certainly has one or more DNA bases with a *base accumulation* value equal to the region length itself, i.e., having all the other region bases included within its neighborhood (see panels a, b and c of Supplementary Figure 2). For a region with length equal to or greater than $2*w+1$, instead, the maximum *base accumulation* value is always $2*w+1$, since only the other input region bases at a max distance of w fall into the neighborhood and contribute to the *base accumulation* value (see panels d and e in Supplementary Figure 2).

S2 RESULT COMPARISON OF METHODS TO IDENTIFY DNA HOT ZONES

To find High Occupancy Target (HOT) DNA zones, in the main article, we describe the *binding region* and *overlap* methods. In Supplementary Figure 3 we can appreciate and compare their behaviours and results for different accumulation types when applied to the example dataset reported in the main article Figure 2.

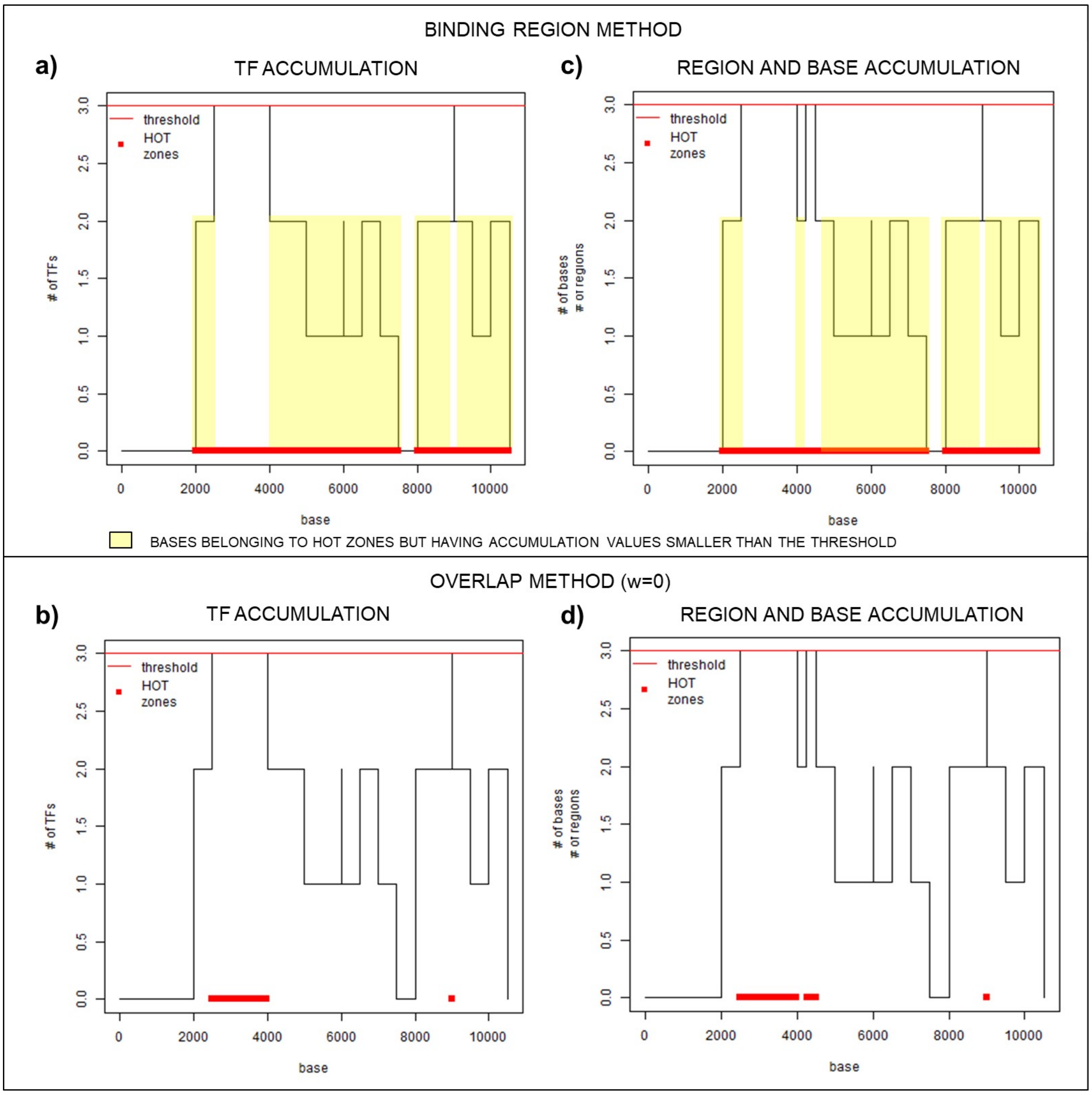

Supplementary Figure 3. HOT zones identified in the example dataset, shown in the main article Figure 2, when considering a null moving window ($w=0$). The *binding region* (a, c) and *overlap* (b, d) methods are compared using *TF accumulation* (a, b), *region* and *base accumulation* (c, d). A threshold of 3.0 is found by the top 1% strategy in all these cases. Notice that no DNA base with accumulation smaller than the threshold is defined as belonging to a HOT zone by the *overlap* approach.

S3 DETAILS OF THE EVALUATION ON PUBLISHED DATA

Here, we provide further details of the comparative analysis between the results of the two presented methods (*binding region* and *overlap*) and those from Chen et al. (2014).

The dataset under exam included 612 samples of 159 different TFs from 90 cell lines, as the original data in Chen et al. (2014), and was analyzed all together, without dividing it by cell lines, as in Chen et al. (2014). Due to unavailability of the exact same data evaluated in that work, we selected the hg19 ENCODE ChIP-seq processed files listed in Chen et al. (2014) from the Anshul Kundaje personal site at the Broad Institute[1] (where they were uploaded on August 3rd, 2012). Yet, some small differences appear in the available data with respect to the original ones, starting from the number of input PEAK regions, which are 11,677,623 in Chen et al. (2014) and 11,696,474 in the dataset we could find; this must be considered in all comparisons of the collected results.

The dataset has been carefully evaluated to assess the distributions of TFs across cell lines and chromosomes, which are reported in panels a) and b) of Supplementary Figure 4. The TFs were well-distributed along chromosomes; accordingly, the accumulation vector (for any accumulation type) spanned over the entire genome. Conversely, as shown in Supplementary Figure 4 c), the majority of cell lines are associated with less than 10 different TFs, while only 6 cell lines are characterized by a wide amount of distinct TFs (from over 20 to almost 100). Accordingly, input binding regions, which are very numerous all together, go from a few thousand to more than 1 million for each cell line, with an average of 23,457 regions per TF and cell line.

An example of the HOT zones found in chromosome 21 with the three presented approaches is depicted in Supplementary Figure 5.

For the *binding region* method and the *overlap* method with the suggested ideal moving window semi-width, we also investigated the presence of HOT zones in promoters and CpG islands, similarly to what done in Chen et al. (2014). Towards this aim, we used hg19 annotations of transcription start sites (TSS) and CpG islands from the "HG19_BED_ANNOTATION" public dataset freely available on the public GMQL repository (Masseroli et al., 2019) at `http://www.gmql.eu/`; this dataset includes CpG island annotations originally from the University of California Santa Cruz, and TSS of known protein-coding genes with an Entrez Gene ID as originally available from the GENCODE repository version 10 (Frankish et al., 2019). To obtain the used promoter region annotations, the DNA coordinates of each TSS have been extended by 2000 bases upstream and 1000 bases downstream.

For both the *binding region* and *overlap* methods, we used the number of HOT zones and the number of dense-but-not-HOT zones (having under-threshold accumulation indices) to build contingency matrices based on their distribution within (or outside) promoters and CpG islands (Supplementary Tables 1 and 2). Such contingency matrices were then used to perform six exact Fisher tests (Sprent, 2011) and assess if the probability of observing a HOT zone in promoters and/or CpG islands is non-randomly greater than that of observing a dense-but-not-HOT zone in the same areas. All these tests returned p-values much smaller than the significance threshold of 0.05 (Supplementary Tables 1 and 2).

	**HOT**	**NOT HOT**			P-VALUE	<2.2E-16
**PROM**	**6,064**	**42,988**	49,052		ODDS RATIO	70.61
**NOT PROM**	**1,378**	**688,484**	689,862		95% CI LOWER	66.43
	7,442	731,472			95% CI UPPER	74.65
	**HOT**	**NOT HOT**			P-VALUE	<2.2E-16
**CpG**	**5,958**	**22,733**	28,691		ODDS RATIO	125.07
**NOT CpG**	**1,484**	**708,739**	710,223		95% CI LOWER	117.88
	7,442	731,472			95% CI UPPER	133.25
	**HOT**	**NOT HOT**			P-VALUE	<2.2E-16
**CpG_PROM**	**5,549**	**17,604**	23,153		ODDS RATIO	118.82
**NOT CpG_PROM**	**1,893**	**713,868**	715,761		95% CI LOWER	112.25
	7,442	731,472			95% CI UPPER	125.76

Supplementary Table 1. Contingency matrices and their Fisher test results assessing the significance of observing, in promoters (PROM) and/or CpG islands (CpG), HOT zones traced by the *binding region* method. CI: Confidence interval.

[1] `https://personal.broadinstitute.org/anshul/projects/encode/rawdata/peaks_spp/mar2012/distinct/idrOptimalBlackListFilt/`

S4 DETAILS OF ANALYSIS OF CONSERVED AND EXCLUSIVE HOT ZONES

ChIP-seq data processing is usually specialized in identifying broad domains (covering wider DNA regions), or narrow peaks (limited to local spikes). For our investigation of TF binding regions in the H1-hESC, K562 and MCF-7 cell lines, we collected only ENCODE samples containing ”optimal IDR thresholded peaks”, i.e., NARROW PEAK samples of higher quality according to the Irreproducible Discovery Rate

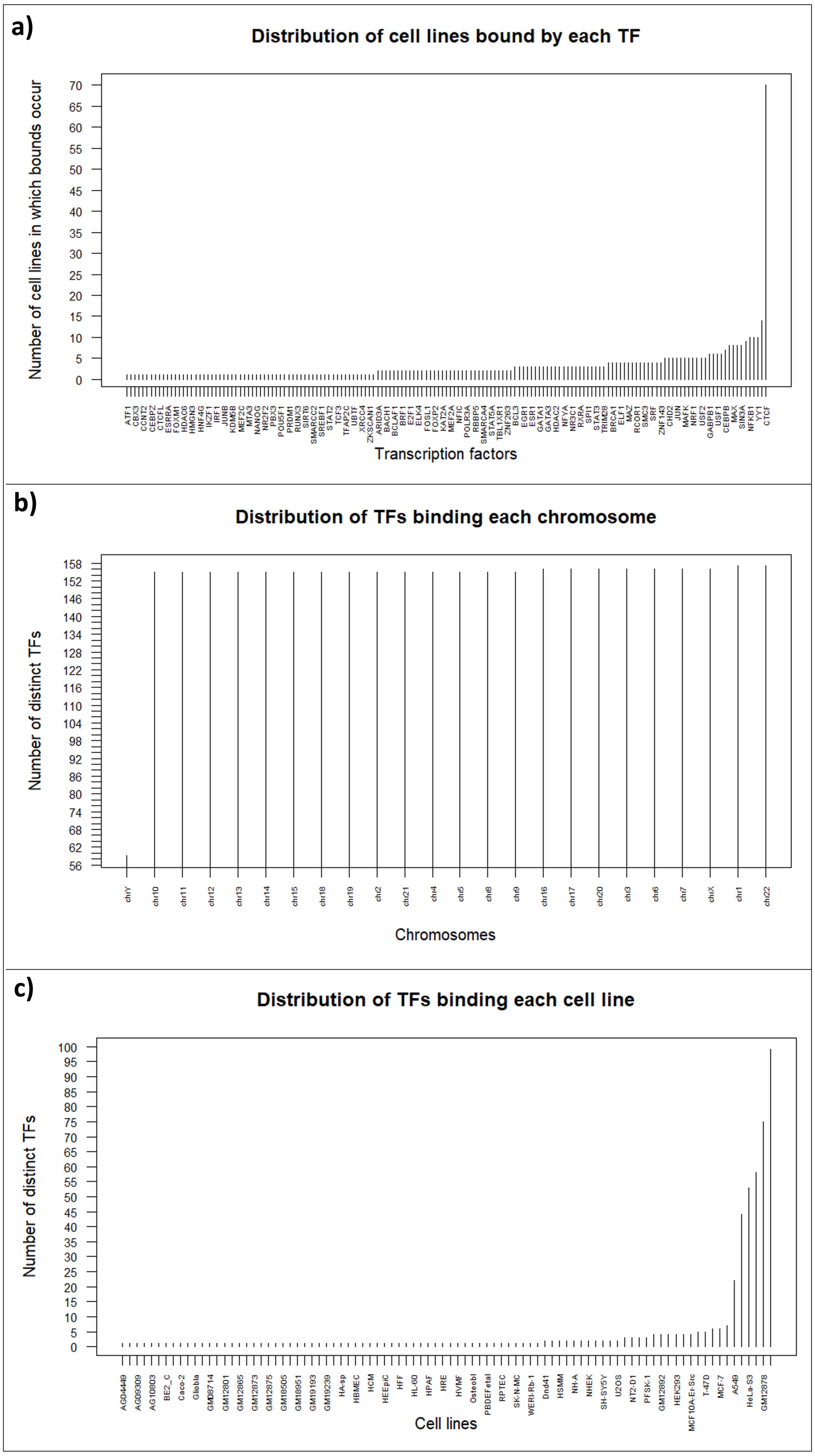

Supplementary Figure 4. Distributions of transcription factors (TFs) binding across cell lines (a, c) and chromosomes (b) in the considered dataset.

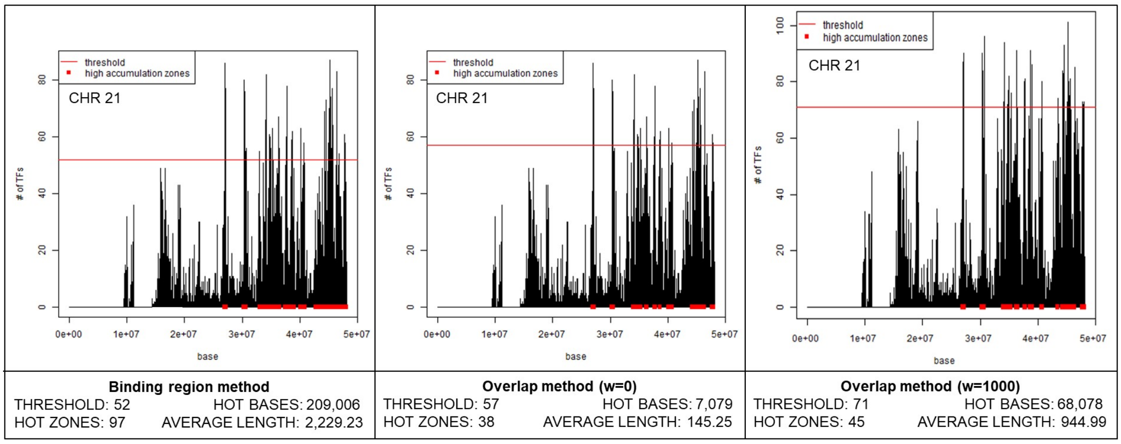

Supplementary Figure 5. *TF accumulation* values and HOT zones identified in chromosome 21 using the 1% threshold and the *binding region* method (left), the *overlap* method with null moving window ($w = 0$) (center), or the *overlap* method with the suggested ideal semi-width ($w = 1000$) for the moving window (right).

	**HOT**	**NOT HOT**			P-VALUE	<2.2E-16
**PROM**	**2,818**	**46,234**	49,052		ODDS RATIO	39.11
**NOT PROM**	**435**	**279,401**	279,836		95% CI LOWER	35.37
	3,253	325,635			95% CI UPPER	43.47
	**HOT**	**NOT HOT**			P-VALUE	<2.2E-16
**CpG**	**2,518**	**26,173**	28,691		ODDS RATIO	39.19
**NOT CpG**	**735**	**299,462**	300,197		95% CI LOWER	36.05
	3,253	325,635			95% CI UPPER	42.70
	**HOT**	**NOT HOT**			P-VALUE	<2.2E-16
**CpG_PROM**	**2,361**	**20,792**	23,153		ODDS RATIO	38.76
**NOT CpG_PROM**	**892**	**304,843**	305,735		95% CI LOWER	35.85
	3,253	325,635			95% CI UPPER	42.04

Supplementary Table 2. Contingency matrices and their Fisher test results assessing the significance of observing, in promoters (PROM) and/or CpG islands (CpG), HOT zones traced by the *overlap* method with $w = 1000$. CI: Confidence interval.

(IDR), which measures the reproducibility of high-throughput experiments (Li et al., 2011). Based on data annotations, we then excluded samples that may contain experimental errors or inaccuracies (i.e., samples with low-quality metrics) as well as those subjected to any pharmacological treatment.

Data processing was performed using RGMQL (Pallotta and Masseroli, 2022; Pallotta et al., 2022), a R/Bioconductor [2] package that provides specialized functions to efficiently extract and process omics data and their metadata, also offering processing outsourcing on remote GMQL engine (Masseroli et al., 2019). For each cell line, input data was filtered to discard outlier binding regions, like those that occupy an abnormally high number of DNA bases (i.e., over the 95 percentile of the region lengths); indeed, these regions are likely due to experimental errors, and their presence can affect the results, especially in terms of accumulation values. Following, replicate samples of experiments having the same target TF were combined together to obtain just one sample for every TF in each cell line.

Investigating six TFs (CTCF, JUN, MAFK, MYC, NRF1, RFX5) whose binding region data are available for each of the considered cell lines, we used our TFHAZ software to compute, along the entire genome, TF dense zones for each cell line; we used all three defined types of accumulation (*TF accumulation*, *region accumulation*, and *base accumulation*) and both a null and an ideal moving window of semi-width 1000 bases. To identify DNA HOT zones, we applied both the *binding region* and *overlap* methods, with both thresholding options (*top k percentage* and *over k standard deviations*), assessing the

[2] `https://bioconductor.org/`

most suitable *k* value for the latter approach. The obtained results are in Supplementary Table 3.

Accumulation		Method	Threshold		H1-hESC HOT zones			K562 HOT zones			MCF-7 HOT zones		
w	type		strategy	*k*	thr	amount	bases	thr	amount	bases	thr	amount	bases
0	TF, R, B	BR	%	1	2	6,449	1,161,974	4	5,265	987,901	3	6,210	1,159,453
0	TF, R, B	BR	STD	1:6	1	73,545	17,557,754	1	108,642	43,487,441	1	130,795	38,675,452
0	TF, R, B	OV	%	1	3	884	130,401	4	5,265	987,901	4	1,480	225,062
0	TF, R, B	OV	STD	1	2	6,449	1,161,974	3	14,987	3,487,316	2	25,301	5,873,950
0	TF, R, B	OV	STD	2	2	6,449	1,161,974	3	14,987	3,487,316	3	6,210	1,159,453
0	TF, R, B	OV	STD	3	3	884	130,401	4	5,265	987,901	3	6,210	1,159,453
0	TF, R, B	OV	STD	4	3	884	130,401	5	1,209	183,201	4	1,480	225,062
0	TF, R, B	OV	STD	5	3	884	130,401	5	1,209	183,201	4	1,480	225,062
0	TF, R, B	OV	STD	6	3	884	130,401	6	161	21,262	5	257	33,643
1000	TF	OV	%	1	3	1,682	2,978,871	5	2,433	4,171,538	4	2,881	5,162,874
1000	R	OV	%	1	4	786	987,004	7	1,698	1,548,226	7	1,096	944,587
1000	B	OV	%	1	805	1,093	1,436,394	2,526	1,604	1,362,618	1,818	1,345	1,389,358
1000	TF	OV	STD	1	2	8,656	16,937,857	3	16,628	36,057,010	2	26,510	58,022,207
1000	R	OV	STD	1	2	11,941	20,637,371	3	19,009	39,477,129	3	14,904	25,926,304
1000	B	OV	STD	1	385	10,665	16,731,198	994	18,303	33,599,790	653	16,362	28,554,465
1000	TF	OV	STD	2	2	8,656	16,937,857	4	7,737	14,925,918	3	8,731	17,183,199
1000	R	OV	STD	2	3	2,965	4,422,225	4	11,201	19,553,992	4	7,752	11,449,105
1000	B	OV	STD	2	525	4,223	6,462,658	1,488	9,692	14,269,610	954	8,696	13,215,202
1000	TF	OV	STD	3	3	1,682	2,978,871	5	2,433	4,171,538	4	2,881	5,162,874
1000	R	OV	STD	3	3	2,965	4,422,225	6	3,275	3,717,855	5	4,033	4,984,173
1000	B	OV	STD	3	666	2,456	3,420,200	1,983	4,252	4,787,867	1,250	4,647	6,148,596
1000	TF	OV	STD	4	3	1,682	2,978,871	6	379	632,057	5	698	1,123,074
1000	R	OV	STD	4	4	786	987,004	7	1,698	1,548,226	6	2,149	2,176,705
**1000**	**B**	**OV**	**STD**	**4**	**806**	**1,092**	**1,434,460**	**2,478**	**1,739**	**1,529,492**	1,557	2,491	2,838,688
1000	TF	OV	STD	5	4	273	449,808	7	-	-	5	698	1,123,074
1000	R	OV	STD	5	4	786	987,004	8	850	632,473	7	1,096	944,587
**1000**	**B**	**OV**	**STD**	**5**	946	621	734,597	2,972	595	419,034	**1,858**	**1,258**	**1,264,581**
1000	TF	OV	STD	6	4	273	449,808	8	-	-	6	139	190,696
1000	R	OV	STD	6	5	198	231,067	9	399	279,372	8	545	397,778
1000	B	OV	STD	6	1,087	296	345,626	3,467	177	108,233	2,159	631	582,567

Supplementary Table 3. Comparison of results from the DNA HOT zone analysis of three ENCODE datasets regarding H1-hESC, K562 and MCF-7 cell lines. For both a null ($w = 0$) and an ideal ($w = 1000$) moving window, we assessed the three types of accumulation (TF: *TF accumulation*, R: *region accumulation*, B: *base accumulation*) and applied both the *overlap* (OV) and *binding region* (BR) methods using the thresholding strategies *top k percentage* (%) with $k = 1$ or *over k standard deviations* (STD) for different *k* values. For each cell line, the computed HOT zone threshold value (thr) and the obtained number of HOT zones (amount) and of their total DNA bases (bases) are reported. The values corresponding to the preferred approach and thresholding option, as reported in the main article Table 3, are in bold.

While the *binding region* method appears less suitable to trace HOT zones when dealing with a reduced set of TFs, the *overlap* method provides good results, using either the *top* 1% threshold or an *over k standard deviations* thresholding with a sufficiently high *k* value. Particularly, with few TFs the use of the *base accumulation* together with a moving window appears as the best option to be combined with the *overlap* method to identify DNA HOT zones. In fact, given the limited ranges of accumulation values obtained for both the *TF accumulation* and *region accumulation*, with few TFs, these accumulation types can make it harder to find a threshold to properly identify the DNA HOT zones: a lot of DNA bases can be indeed associated with accumulation values equal to or greater than a threshold value (or lower than the next threshold value) and HOT zones result much wider (or much narrower and much less).

For a biologically-relevant further comparison of the results for the three cell lines, we focused on the HOT zones obtained using the most promising approach (*overlap* method on *base accumulation* with an ideal moving window) and thresholding option (*over k standard deviations*): these are highlighted in bold in Supplementary Table 3. From such selected results, we distinguished HOT zones conserved (i.e., overlapping, although partially, with at least a HOT zone in both the other cell lines) or exclusive for each cell line. Supplementary Figure 6 reports a Venn diagram including the amounts of conserved, exclusive and shared (in two out of the three cell lines) HOT zones from each of the three cell lines. Notice that such amounts can be slightly different for each cell line because the boundaries of the HOT zones in each cell line depend on the local accumulation of the underlying bases and on how the consecutive bases are grouped by the overlap method in the given cell line dataset.

Then, to characterize the HOT zones exclusive of just one cell line, we used the annotation database from the University of California Santa Cruz for the Genome Reference GRCh38[3]: specifically, we traced all the genes having gene body or promoters (defined as 2000 bases upstream and 1000 bases downstream

[3]available as R/Bioconductor Annotation Package at `https://bioconductor.org/packages/release/data/annotation/html/TxDb.Hsapiens.UCSC.hg38.knownGene.html`

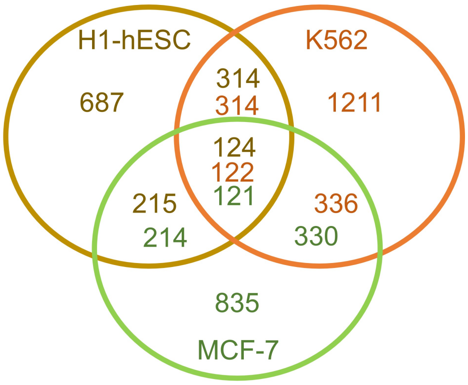

Supplementary Figure 6. Venn diagram with all the conserved among the three, shared in two out of three, and exclusive HOT zones for each cell line.

the gene transcription start sites) intersecting a cell line-exclusive HOT zone. However, some of the so-found genes overlapped multiple exclusive HOT zones of different cell lines; thus, after a comparative evaluation, for each cell line, we selected only those genes related exclusively to the HOT zones of that cell line. In this way, we obtained 2,193 genes for H1-hESC, 1,873 for K562 and 1,309 for MCF-7 cell line.

Functional enrichment analysis of each of the obtained cell line-exclusive gene lists was performed with g:Profiler (Reimand et al., 2007) and with Enrichr (Chen et al., 2013), to evaluate the enrichment in Gene Ontology and/or pathway annotations from multiple databases. A p-value threshold of 0.05 was used with alternative multiple testing correction options (Benjamini-Hochberg FDR, or g:SCS algorithm (Reimand et al., 2007)) to better assess statistical significance, taking into account also the unevenly distributed hierarchical structure of the annotation terms. This functional investigation of HOT zone-associated genes is indeed a key aspect to study and compare active genomic regions, highlighting relevant biological traits as well as contributions to biological processes and gene expression regulation in different biological conditions. Relevant results are discussed in the main article subsection *Comparative results and experimental evidences* of the Section *Analysis of conserved and exclusive DNA HOT zones*.

REFERENCES

Chen, E. Y., Tan, C. M., Kou, Y., Duan, Q., Wang, Z., Meirelles, G. V., Clark, N. R., and Ma'ayan, A. (2013). Enrichr: interactive and collaborative HTML5 gene list enrichment analysis tool. *BMC Bioinformatics*, 14(1):1–14.

Chen, R. A.-J., Stempor, P., Down, T. A., Zeiser, E., Feuer, S. K., and Ahringer, J. (2014). Extreme HOT regions are CpG-dense promoters in C. elegans and humans. *Genome Research*, 24(7):1138–1146.

Frankish, A., Diekhans, M., Ferreira, A.-M., Johnson, R., Jungreis, I., Loveland, J., Mudge, J. M., Sisu, C., Wright, J., Armstrong, J., et al. (2019). GENCODE reference annotation for the human and mouse genomes. *Nucleic Acids Research*, 47(D1):D766–D773.

Li, Q., Brown, J. B., Huang, H., and Bickel, P. J. (2011). Measuring reproducibility of high-throughput experiments. *The Annals of Applied Statistics*, 5(3):1752–1779.

Masseroli, M., Canakoglu, A., Pinoli, P., Kaitoua, A., Gulino, A., Horlova, O., Nanni, L., Bernasconi, A., Perna, S., Stamoulakatou, E., et al. (2019). Processing of big heterogeneous genomic datasets for tertiary analysis of Next Generation Sequencing data. *Bioinformatics*, 35(5):729–736.

Pallotta, S., Cascianelli, S., and Masseroli, M. (2022). RGMQL: scalable and interoperable computing of heterogeneous omics big data and metadata in R/Bioconductor. *BMC Bioinformatics*, 23(1):1–28.

Pallotta, S. and Masseroli, M. (2022). RGMQL: GenoMetric Query Language for R/Bioconductor. `https://www.bioconductor.org/packages/release/bioc/html/RGMQL.html`. R/Bioconductor package version 1.18.0.

Reimand, J., Kull, M., Peterson, H., Hansen, J., and Vilo, J. (2007). g:Profiler — a web-based toolset for functional profiling of gene lists from large-scale experiments. *Nucleic Acids Research*, 35(Web Server issue):W193–W200.

Sprent, P. (2011). *Fisher Exact Test*, pages 524–525. Springer, Berlin, Heidelberg.
